# Association of Antipsychotic Polypharmacy VS Monotherapy with psychiatric re-hospitalization among adults with schizophrenia followed By the ACT TEAM in Bronx

**DOI:** 10.1192/j.eurpsy.2025.1557

**Published:** 2025-08-26

**Authors:** O. Alli-Balogun, U. Anyeji, S. Gunturu

**Affiliations:** 1 Psychiatry, Bronxcare Health System, New York, United States

## Abstract

**Introduction:**

The ACT team consists of mental health professionals with a wide range of expertise, including psychiatrists, psychologists, nurses, social workers, and peer specialists. The goal of ACT is to help patients achieve and maintain stability in the community, and to reduce the need for hospitalization and other forms of institutional care and reduce the barriers to care by bringing the care to the Patient.

Upon discharge from the Psychiatric Hospital Patients are often sent home with more than one antipsychotic oral medication and are at times on two long-acting intramuscular preparations.

This is an example of polypharmacy which refers to the concurrent use of multiple medications to treat a single patient, polypharmacy is often used to manage patients with complex mental health conditions who may require multiple medications to address their symptoms.

**Objectives:**

The AIM of the study is to determine the number of patients followed by the ACT team in the Bronx who are on 2 or more antipsychotics as compared to those on monotherapy who were re-hospitalized within 1 year of discharge.

**Methods:**

This is a retrospective study on patients who are followed by Bronx ACT team affiliated with the Institute for Community Living (ICL) in NYC from February 2022 to February 2023. The study compared the number of re- hospitalizations of patients on 2 or more Antipsychotics as compared to those on Monotherapy from February 2022- February 2023. were a thorough review of charts of the 68 Clients being followed by the ACT team was done.

**Results:**

Out of 68 patients being followed by the ACT team; 9 Patients were on 2 antipsychotics/ LAI (Long Acting Injectable) of which 5 patients were on 2 oral antipsychotics, 1 Patient on 3 oral antipsychotics, and 3 Patients on two long-acting intramuscular depot preparations of which five 5(3 for psychiatric related issues and 2 for medical reasons) out of 9 (55.55%) of this population were hospitalized in the 12-month reporting period of February 2022 to February 2023. As compared to 27 (45.7%) patients on monotherapy who were also hospitalized in the last 12-month reporting period.

All remaining 32 (54.2%) clients on monotherapy had no hospitalizations in the 12 months reporting period.

**Conclusions:**

Patients who warrant the need to be followed by The ACT are majorly those who require intensive follow-up in the community and are mostly non-adherent with their medications and prescribing multiple antipsychotics that usually not taken should be carefully considered.

Due to the nature of the study no definite conclusion could be drawn regarding causality between APP and hospitalizations within the 12- month reporting period.

**Disclosure of Interest:**

None Declared

